# Energy acquisition strategy for reproduction in a semelparous squid

**DOI:** 10.1186/s12983-022-00473-w

**Published:** 2022-11-16

**Authors:** Dongming Lin, Na Zang, Kai Zhu, Gang Li, Xinjun Chen

**Affiliations:** 1 College of Marine Science, Shanghai Ocean University, Shanghai, 201306 China; 2 Key Laboratory of Sustainable Exploitation of Oceanic Fisheries Resources, Ministry of Education, Shanghai, 201306 China; 3 National Engineering Research Center for Oceanic Fisheries, Ministry of Science and Technology, Shanghai, 201306 China; 4 Key Laboratory of Oceanic Fisheries Exploration, Ministry of Agriculture and Rural Affairs, Shanghai, 201306 China; 5 Zhejiang Marine Fisheries Research Institute, Zhoushan, 316021 China

**Keywords:** Squid, Energy demand, Acquisition strategy, Reproduction, Semelparity

## Abstract

**Background:**

Energy demand for reproduction leads to a wide diversity of foraging and life-history strategy among wild animals, linking to a common objective to maximize reproductive success. Semelparous squid species in particular can use up to 50% of the total energy intake for reproduction. However, the energy acquisition strategy for reproduction is still a controversial issue regarding whether the squid shift in diet ontogenetically. Here we used Argentinean shortfin squid (*Illex argentinus*) as a case study to investigate the strategy of energy acquisition for reproduction, by analyzing energy density of the squid’s reproductive tissues including ovary, nidamental glands and oviduct eggs, and stable isotopes and fatty acids of the squid’s ovary.

**Results:**

The reproductive energy (the sum of the energy accumulated in ovary, nidamental glands and oviduct eggs) increased significantly with maturation. The ovary nitrogen stable isotopes (δ^15^N) showed a significant increase with maturation, but the increase by maturity stage was not equal to the typical enrichment of about 3‰ per trophic level. Isotopic niche width showed an increasing trend with maturation, and isotopic niche space exhibited greater overlap at advanced maturity stages. The relative amounts of 16:0, 20:5n3 and 20:4n6 in the ovary, tracing for carnivores and top predators, increased after the onset of maturation. The overall fatty acid profiles of the ovary showed significant differences among maturity stages, but obvious overlaps were found for mature squids. Mixed-effects model results revealed that reproductive energy was positively correlated with δ^15^N values. The reproductive energy was also positively related to the relative amounts of 18:0 and 20:4n6, respectively tracing for herbivores and top predators.

**Conclusions:**

Our results validate that the squid shifts to feed on higher trophic prey for reproduction as energy demand increases once maturation commences. However, the squid does not shift feeding habits at a trophic level but instead broadens prey spectrum, coupled with increasing intake of higher trophic prey items, to meet the energy demand for reproduction. Such energy acquisition strategy may be selected by the squid to maximize reproductive success by balancing energy intake and expenditure from foraging, warranting future studies that aim to clarify such strategy for reproduction among semelparous species.

**Supplementary Information:**

The online version contains supplementary material available at 10.1186/s12983-022-00473-w.

## Background

Energy demands scale differently with species growth and diet, and particularly the follow of reproductive energy leads to a wide diversity of life-history among wild animals [1]. Behavioral and life-history strategies for an animal are selected for or against over evolutionary time to acquire energy from prey and manage energy budgets [2]. At a coarse scale, these strategies are centered on maximizing reproduction to leave as many descendants as possible [3, 4]. Indeed, the most substantial metabolic expenditure for many species occurs during reproduction [5, 6]. This is particularly a case for semelparous animals, which on average, invest much more energy into a single reproductive episode before death than iteroparous animals that reproduce in multiple cycles over the course of their lifetime [1, 7]. However, due to the conservation of matter and energy where resources allocated to one function are not available for others, animals must face a fundamental trade-off in allocating limited resources to a basic life-history function such as survival and reproductive investment [8]. Driven by the trade-off, energy demand and allocation to reproduction appear to interact with the physiological and metabolic constraints of an animal to give rise to the remarkable diversity of foraging and life-history strategies observed in nature [9].

Squid are one of the most important cephalopod species [10], playing an essential role in the trophic webs of marine ecosystems [10] and global production of marine fisheries [11]. They are well-known for having a monocyclic life-history, characterized by a short lifespan, fast growth and semelparous reproduction [10]. During maturation they usually allocate large amounts of energy for reproduction that can amount to 50% of their body mass [12]. This allocation of energy influences the number and success of offspring, and ultimately determines population size and stability over time [13, 14]. To meet energy demands for reproduction, these species likely evolved an ontogenetic shift in diet, coupled with opportunistic and voracious foraging behavior [10, 15]. For example, Ommastrophid squids shift in diet from crustaceans to fish as they grow, in which there is a marked increase in the proportion of fish [16–18]. Since prey items such as fish occupy higher trophic positions, they have greater nutrient content [19] and greater energy density [20, 21]. It is expected that the uptake of such prey items would improve metabolic energy and support better life-history functions [22]. However, higher trophic animals generally have better predator avoidance, which in turn increases the energy spent by the consumers on hunting [23]. Such energy expenditures should theoretically be saved when the consumer forages for small prey species to gain a comparative energy resource [24]. It is also assumed that the reason why squid species shift feeding habits ontogenetically is because they become better at hunting as they grow rather than the increased need of energy for reproduction [15, 25]. It is still unclear how reproductive energy needs are met for semelparous animals, which is important for understanding their life-history.

We used Argentinean shortfin squid *Illex argetinus* as the model system to investigate the energy acquisition strategy for the semelparous squids during maturation. This species represents a typical semelparous squid species, and is well documented for its fast growth, short life span and large amount of energy drawn for reproduction once maturation commences [26]. Furthermore, this species not only plays a key role as a transient “biological pump” in the southwest Atlantic ecosystem [27], but it is also one of the most important commercial species in the world [11]. In this study, we estimated the reproductive energy of *I. argentinus* by applying the technique of tissue energy density, and evaluated the energy acquisition strategy for reproduction using biochemical markers of stable isotopes and fatty acid profiles. The technique of tissue energy density has been successfully applied to investigate reproductive energy accumulation during maturation for *I. argentinus* [28, 29], as well as squid species *Dosidicus gigas* [30] and *Sthenoteuthis oualaniensis* [31]. Generally, carbon (δ^13^C) and nitrogen (δ^15^N) stable isotopes are transferred from dietary sources to consumers in a predictable manner [32], where δ^15^N is typically enriched by about 3‰ per trophic level, and δ^13^C shows little change due to trophic transfer [32, 33]. Thus, the δ^15^N of a given consumer can be used to reflect the variations of prey, and the isotopic space can be used to indicate feeding niche widths of the predator [32]. Fatty acids are essential components of all living organisms and passed with little to no modification from prey to predator, which make them useful tracers of diets and marine food-web structure [34]. Analyzing the stable isotopes and fatty acids of reproductive tissues can infer the energy acquisition strategy for reproduction for the squid.

**Fig. 1:**
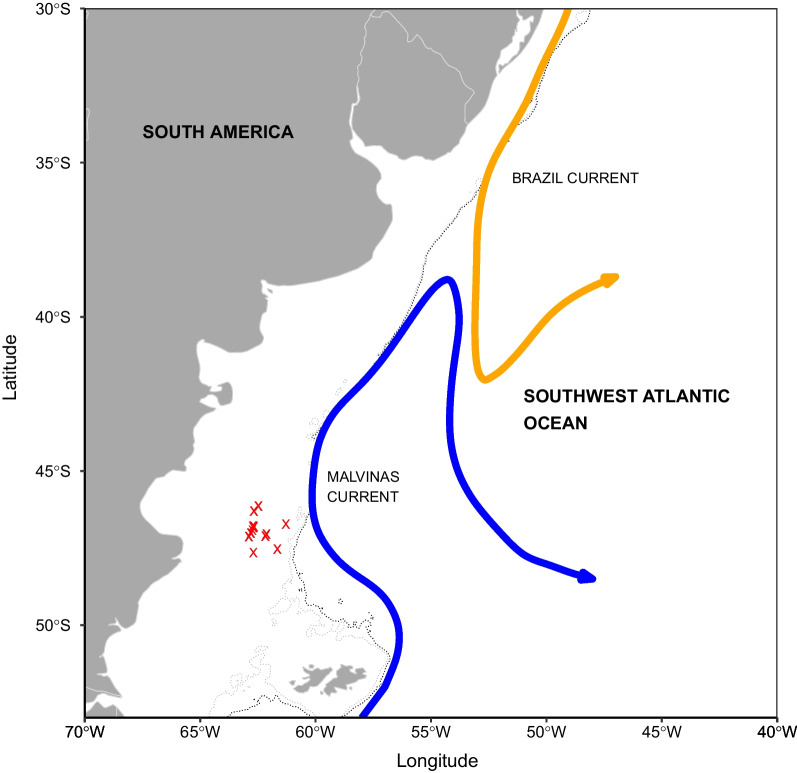
Sampling region and sampling stations in the Southwest Atlantic Ocean. Red cross (x) presents the sampling station. Grey and black dotted lines represent the isobaths 200 m and 300 m, respectively

We randomly collected the Argentinean shortfin squid *I. argetinus* specimens from the catches of commercial jiggers on the high seas area of the Patagonian shelf in the southwest Atlantic Ocean (61° 09′ W ~ 62° 53′ W, 46° 08′ S ~ 47° 51′ S; Fig. 1) from January to March 2019. We aimed to investigate the strategy of energy acquisition when energy demand for reproduction is the greatest for the squid [28, 35]. We particularly aimed to clarify whether the squid exclusively feeds on prey items at a higher trophic or lower trophic position, or uses a combination, to meet reproductive energy demands. To further clarify the energy acquisition strategy, we also used linear mixed-effects models [36] to evaluate the relationship between reproductive energy and stable isotopic values and fatty acid profiles. These analyses will allow for a more comprehensive understanding of the life-history strategy in squid species and demonstrate the potential application of these methods to other semelparous animals in nature.

## Results

The reproductive system of *I. argetinus* increases dramatically once maturation commences [26], therefore we selected 68 female specimens at maturity stages from physiologically maturing (stage III) to spawning (stage VI) after the scales proposed by ICES [37] and Lin et al. [38], for reproductive energy analysis, carbon (δ^13^C) and nitrogen (δ^15^N) stable isotope analysis, and fatty acids analyses. The sample size was 12 at stage III (physiologically maturing), 15 at stage IV (early physiologically mature), 15 at stage V (late physiologically mature), 15 at stage VI (functionally mature), and 11 at stage VII (spawning). The squids' measured dorsal mantle length ranged from 184 to 262 mm and weighed body mass from 129 to 374 g. Both mantle length and body weight exhibited unimodal distribution (Additional file 1: Fig. S1a–b), indicating that the squids were from a single population stock [26]. Body weight was significantly correlated with mantle length, with a power function *W* = 0.00013 × *L*^2.66^ (*r*^*2*^ = 0.67, *p* < 0.05; Additional file 1: Fig. S1c). There were no significant differences in mantle length or body weight among maturity stages (mantle length, *F* = 0.92, *p* = 0.46; body weight, *F* = 1.10, *p* = 0.37; Additional file 1: Fig. S2).

### Reproductive energy

**Fig. 2:**
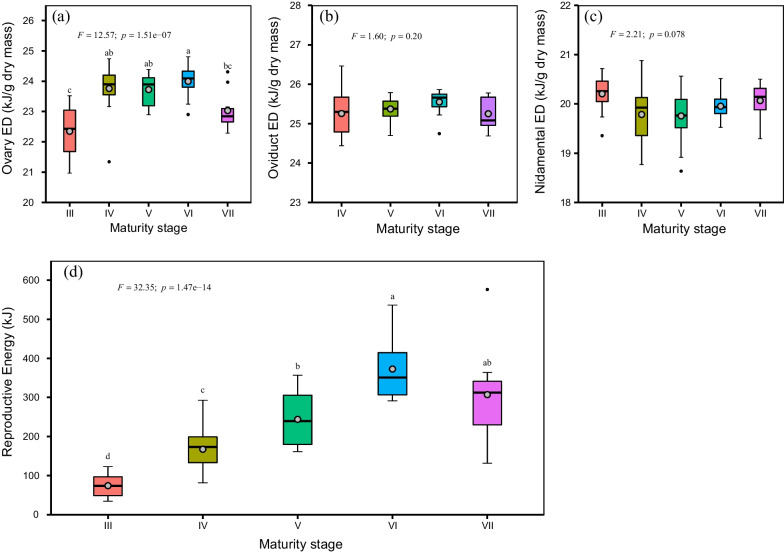
Distribution of energy density and reproductive energy by maturity stages for Argentinean shortfin squid. **a**, ovary energy density; **b**, energy density of oviduct eggs; **c**, energy density of nidamental glands; **d**, reproductive energy, estimated as the sum of the energy accumulated in ovary, nidamental glands and oviduct eggs. The boxplot horizontal line and grey solid point respectively denote the median and mean, while upper and lower hinges respectively represent the 25th and 75th percentiles. The maturity stages were followed the scale proposed by ICES [37] and Lin et al. [38]: III physiologically maturing, IV–V, physiologically mature, VI, functionally mature, and VII, spawning. The sample size by maturity stage was 12 at stage III, 15 at stage IV, 15 at stage V, 15 at stage VI, and 11 at stage VII

The energy density of ovary, nidamental glands and oviduct eggs (henceforth “eggs”) was determined using an automatic isoperibol calorimeter. The average energy density over all maturity stages was 23.44 ± 0.89 kJ/g dry mass for ovaries, 25.38 ± 0.40 kJ/g dry mass for eggs, and 19.93 ± 0.48 kJ/g dry mass for nidamental glands, respectively. Energy density in the ovary increased significantly with maturation (*F* = 12.57, *p* < 0.05). Greater energy density within ovaries was observed at stages IV-VI; while both eggs and nidamental glands remained relatively constant (Fig. 2a–c). The reproductive energy, estimated as the sum of the energy accumulated in the ovary, nidamental glands and eggs, increased significantly with maturation (*F* = 30.31, *p* < 0.05), with a five-fold increase seen from stage III (74.12 ± 31.07 kJ) to stage VI (372.79 ± 82.85 kJ) (Fig. 2d).

### Stable isotopes

**Fig. 3:**
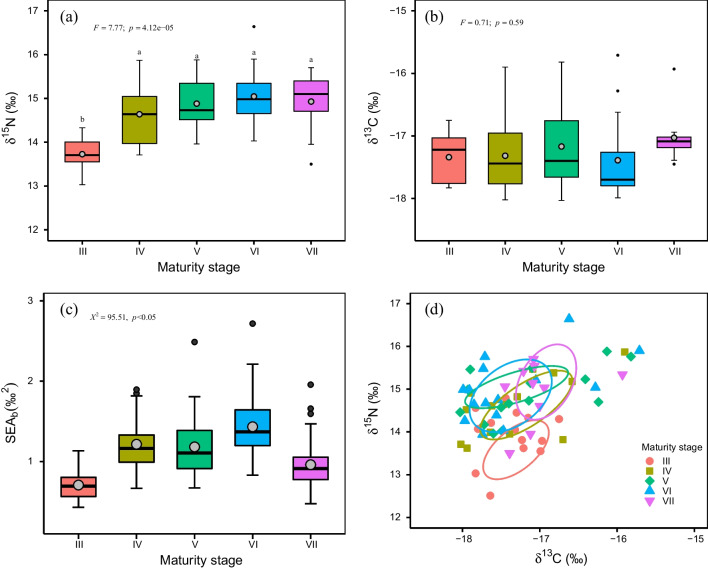
Stable isotopes and estimates of isotopic niche area for Argentinean shortfin squid. Isotopic values were determined from the squid’s ovary. **a**, nitrogen isotope ratio distribution with maturation; **b**, carbon isotope ratio distribution with maturation; **c** Bayesian standard ellipse area (SEA_b_) estimates for each maturity stage. The grey solid circles and horizontal lines in the boxes indicating the means and medians; **d** Corrected standard ellipse area (SEA_c_) for each maturity stage with 40% Bayesian credible intervals. The boxplot horizontal line and grey solid point respectively denote the median and mean, while upper and lower hinges respectively represent the 25th and 75th percentiles. Maturity stage scale and sample size as presented in Fig. 2

The δ^15^N values of the ovary were determined from 13.03 to 16.64 ‰, and exhibited a significant increase trend with maturation (*F* = 7.77, *p* < 0.05), reaching a peak value of 15.05 ± 0.68 ‰ at stage VI (Table 1; Fig. 3a). However, the increase of δ^15^N values by maturity stage was not equal to a typical enrichment of about 3‰ per trophic level [33]. The δ^13^C values varied from − 18.03‰ to − 15.71‰, but did not vary significantly between maturity stages (*F* = 0.71, *p* = 0.58), with an overall mean value of − 17.26 ± 0.59 ‰ (Table 1; Fig. 3b).

**Table 1:** Stable isotopic metrics of Argentinean shortfin squid

Maturity stage	δ15N(‰)		δ13C(‰)		Isotopic niche width	
	Range	Mean ± SD	Range	Mean ± SD	SEAc	Non-overlap SEAc proportion
III	13.03 to 14.33	13.73 ± 0.40	− 17.83 to − 16.75	− 17.34 ± 0.40	0.57	
						0.03
IV	13.71 to 15.87	14.64 ± 0.66	− 18.02 to − 15.9	− 17.32 ± 0.60	1.06	
						0.59
V	13.96 to 15.88	14.88 ± 0.61	− 18.03 to − 15.82	− 17.17 ± 0.70	1.18	
						0.46
VI	14.03 to 16.64	15.05 ± 0.68	− 17.99 to − 15.71	− 17.39 ± 0.68	1.32	
						0.35
VII	13.50 to 15.70	14.93 ± 0.71	− 17.45 to − 15.93	− 17.03 ± 0.42	1.00	
Pooled	13.03 to 16.64	14.69 ± 0.75	− 18.03 to − 15.71	− 17.26 ± 0.59		

SEAc, corrected standard ellipse area; non-overlap SEAc proportion, overlap as proportion of the sum of the non-overlapping SEAc between two consecutive maturity stages. The non-overlap SEA_c_ proportion ranges from 0 (zero overlap in the isotopic niche widths between groups) to 1 (complete overlap in the isotopic niche widths between groups)

Both δ^15^N and δ^13^C values showed greater variability (standard deviations [SD]) with maturation (Table 1). The corrected standard ellipse area (SEA_c_), calculated as the standard ellipse area of isotopic covariance matrix corrected by sample size using Bayesian approach [39], ranged from 0.57 at stage III to 1.32 at stage VI (Table 1) and revealed the increase of trophic niche width with maturation. In addition, the Bayesian standard ellipse area (SEA_b_), a posterior estimate of isotopic matrix using the Bayesian approach [39], was significantly different between maturity stages (Kruskal–Wallis, *χ2* = 95.51, *p* < 0.05; Fig. 3c), further revealing the differences in the isotopic niches for the squids between maturity stages. The overlap in the isotope niches was estimated as the ratio of overlap proportion and the sum of the non-overlapping area between each consecutive SEA_c_ [39]. This ellipse plots with 40% Bayesian credible intervals was almost separated between stages III and IV however, was obvious overlap after reaching stage IV, with the largest overlap occurring between stages IV and V (Table 1). This observation was confirmed by the overlap of the SEA_c_ of isotopic data for each maturity stage (Fig. 3d).

### Fatty acid profiles

**Fig. 4:**
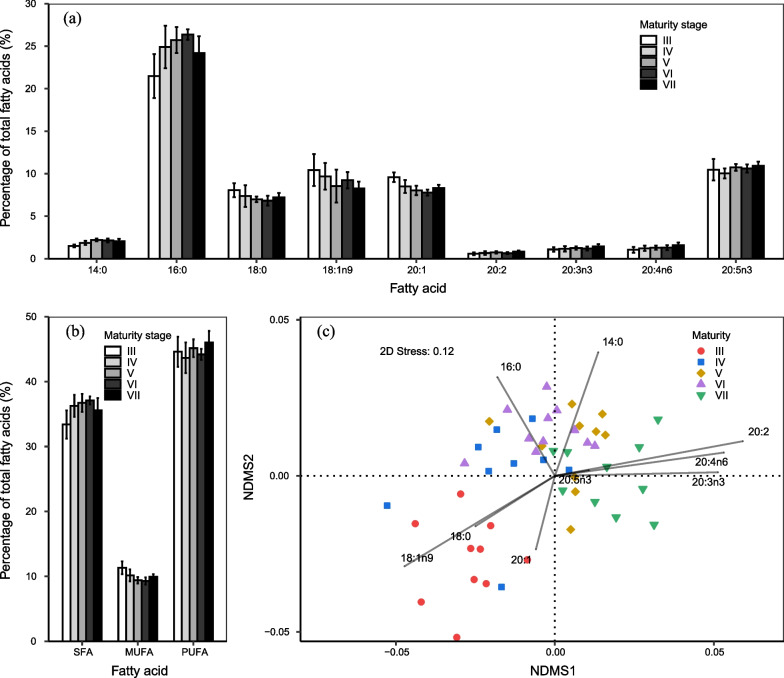
Fatty acid profiles of Argentinean shortfin squid. Values were determined from the squid’s ovary. **a**, relative amounts (mean ± SD) of selected fatty acids with maturation; **b**, relative amounts (mean ± SD) of main fatty acid classes. SFA, saturated fatty acids; MUFA, monounsaturated fatty acids; PUFA, polyunsaturated fatty acids; **c** non-metric multidimensional scaling (NMDS) of the fatty acid composition with maturation with overlaid vectors of individual fatty acids detected significant differences between stages. Maturity stage scale and sample size as presented in Fig. 2

A total of 28 fatty acids (FAs) were determined in the squid’s ovaries, 14 of which had relative mean values greater than 0.5% (Additional file 1: Table S1). Total content of fatty acids (total FAs) increased significantly from stages III–IV, and the 14 fatty acids accounted for 90.06% ~ 97.97% of total FAs, with an average 97.04 ± 1.34%. Nine out of the 14 FAs showed significant differences in the relative amounts between maturity stages, where an increasing trend was found for 14:0 (*F* = 20.41, *p* < 0.05), 20:2 (*F* = 4.17, *p* = 0.006), 20:3n3 (*F* = 3.08, *p* = 0.024), 20:4n6 (*F* = 4.48, *p* = 0.004) and 20:5n3 (*F* = 3.20, *p* = 0.028), a decreasing trend for 18:0 (*F* = 4.31, *p* = 0.005), 18:1n9 (*F* = 3.53, *p* = 0.013) and 20:1 (*F* = 18.37, *p* < 0.05), and an increase for 16:0 from stages III–VI (*F* = 18.18, *p* < 0.05), followed by a decrease at stage VII (Fig. 4a; Additional file 1: Table S1). The main fatty acid classes, namely saturated fatty acids (SFA), monounsaturated fatty acids (MUFA), and polyunsaturated fatty acids (PUFA), were also significantly different in the relative amounts between maturity stages. SFA increased from stages III–VI (*F* = 8.87, *p* < 0.05), PUFA showed an increasing trend with maturation (*F* = 3.56, *p* = 0.035), while MUFA decreased from stages III–VI (*F* = 14.15, *p* < 0.05) (Fig. 4b; Additional file 1: Table S1).

The analysis of similarity (ANOSIM) revealed that the overall FA profiles showed significant differences among maturity stages (global *R*-value = 0.35, *p* = 0.001). However, the significant differences were only found between stages III and IV (global *R*-value = 0.30, *p* = 0.004) and between stages VI and VII (global *R*-value = 0.37, *p* = 0.001) (Table 2). Non-metric multidimensional scaling (NMDS) results showed a large amount of overlap in the overall FA profiles between stages IV, V and VI, and obvious segregations between stages III and IV and between stages VI and VII (Fig. 4c). FAs 18:0, 18:1n9 and 20:1 exhibited the highest values at stage III, 14:0 and 16:0 at stages V and VI, and 20:2, 20:3n3 and 20:4n6 at stage VII (Fig. 4c; Additional file 1: Table S1).

**Table 2:** Results of analysis of similarities (ANOSIM) for the differences in fatty acid composition between two consecutive maturity stages for Argentinean shortfin squid

Maturity stage	R value	*p*
III versus IV	0.30	0.004
IV versus V	0.14	0.046
V versus VI	0.03	0.228
VI versus VII	0.37	0.001
Pooled	0.35	0.001

The R value of ANOSIM ranges from − 1 to 1, with value close to 1 indicating high difference between groups, while value close to 0 indicates high similarity

### Relationship between reproductive energy and nitrogen isotopes and fatty acids

**Fig. 5:**
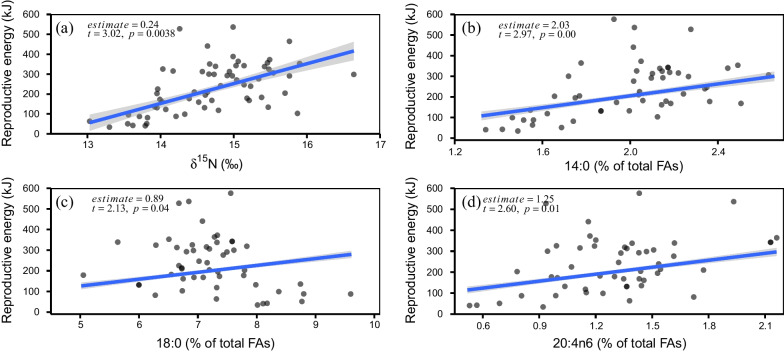
Reproductive energy relation to nitrogen stable isotope ratio and selected fatty acids for Argentinean shortfin squid. Isotopic values and fatty acid profiles were determined from the ovary of Argentinean shortfin squid. Relationships between reproductive energy and nitrogen isotope ratio (**a**), relative amounts of 14:0 (**b**), 18:0 (**c**) and 20:4n6 (**d**). Estimate in the top-left corner depicts average fixed-effects exponent value; blue lines depict average model fits, with 95% confidence intervals in grey shading

The relationships between reproductive energy and δ^15^N and FAs were separately carried out by applying linear mixed-effects models (LMMs), where mantle length and sea surface chlorophyll-*a* concentration were included as the explanatory variables, and maturity stage was included as a random effect to account for the potential effect caused by sexual development. Results from the LMMs revealed that the reproductive energy was positively correlated with δ^15^N values (*t*-value = 3.02, *p* = 0.004; Fig. 5a; Additional file 1: Table S2). Similarly, the reproductive energy was also positively related to the relative amounts of 14:0 (*t*-value = 2.97, *p* = 0.005), 18:0 (*t*-value = 2.13, *p* = 0.04), 20:4n6 (*t*-value = 2.60, *p* = 0.01) (Fig. 5b–d, Additional file 1: Table S3), where 18:0 is assumed to be the trophic marker for herbivores [40], and 20:4n6 for top predators [41]. These findings suggest that the larger amount of reproductive energy is obtained by increasing intake of higher trophic prey items. However, there was no significant relationship between reproductive energy and mantle length (*p* > 0.05; Additional file 1: Table S2, S3). The reproductive energy was also not significantly correlated with the sea surface chlorophyll-*a* concentration (*p* > 0.05; Additional file 1: Table S2, S3), although the sea surface chlorophyll-*a* concentration in the sampling area varied significantly between weeks (*F* = 850.69, *p* < 0.05; Additional file 1: Figure S3).

## Discussion

### Reproductive energy

The energy density of the squid ovary increased as maturation progressed, while both eggs and nidamental glands were relatively stable throughout sexual maturation. This pattern is consistent with previous research conducted on the same species [28, 29] and other squid species (e.g. *D. gigas* [30]; *S. oualaniensis* [31]). The increase of energy density in the ovary may be related to yolk production during ovarian development [42]. Stable energy density of eggs is an indication of similar quality egg production, which must be of great importance for these semelparous species to maximize reproductive success [10]. Consequently, the reproductive energy increased significantly with maturation (Fig. 1d), most likely in relation to the enlargement of reproductive systems [38]. More importantly, the increase of reproductive energy should be considered as the increase of energy demand for reproduction. *Illex argentinus* has been found to significantly increase energy allocation to reproduction once maturation commences [28, 29]. Many monocyclic species have also been reported to direct large amounts of energy to reproduction at the onset of maturation [12, 43, 44]. This is most likely an evolutionary selection given the semelparous nature of the species and the need to maximize reproductive success.

### Energy acquisition strategy for reproduction

The squid adopts an energy acquisition strategy where individuals feed on higher trophic prey items leading to an increase of energy in reproductive organs during maturation. Such phenomenon is supported by the results of LMMs that were performed the reproductive energy on either the nitrogen isotopes or the selected FA profiles, where the reproductive energy is positively related to δ^15^N values (Fig. 5a), and the relative amounts of carnivorous fatty acid tracer 18:0 and top predator fatty acid tracer 20:4n6 (Fig. 5b–c). Given the fact that energy allocation to reproduction is dramatically increasing once sexual development commences [26], the intake of higher trophic prey items is likely necessary to meet the energetic demand following the hypothesis that prey species at higher trophic position have a greater nutrient content [19].

The squid shifts feeding habits with maturation, especially during the course from stages III–IV, evidenced by the increase trend of δ^15^N values (Fig. 3a). Further evidence can be provided by the fatty acids analyses (Fig. 4). The relative amounts of 16:0 and 20:5n3, dietary tracers for carnivores [40, 45], increase along with maturation, while the relative amounts of 18:0 and 20:1, tracing for herbivores [46], decrease after the onset of maturation. Moreover, the relative amounts of 20:4n6, a tracer for top predator [41], also shows a statistically significant increasing trend throughout sexual maturation. Since consumers are subject to biochemical limitations in biosynthesizing dietary fatty acids [34], it is reasonable to expect that the squid may decrease the intake of prey species at lower trophic level, and turn to higher trophic preys after the onset of maturation. This finding is consistent with the results of stomach content analyses where the squids change in diet composition as they grow, switching from crustaceans at small sizes to fishes and cephalopods at large sizes [47–50].

However, the squid does not shift prey items at a trophic level completely. The enrichment of δ^15^N values between each two consecutive maturity stages is not typically enriched by about 3‰ per trophic level [33]. By contrast, the variance of δ^15^N values at each maturity stage increased with maturation, being the greatest variance occurred just before spawning stage (Table 1). The Bayesian isotopic niche analyses confirmed that the squid at more advanced maturity stage has greater isotopic niche width, indicated by the increase of SEA_c_ and SEA_b_ (Table 1; Fig. 3c). Given the isotopic niche linking to prey [32], these observations indicate that the squid increases prey spectrum to meet energy demands for reproduction [26]. The maturing squids showed larger amounts of herbivorous FA tracers 18:0 and 20:1 [46], while mature and spawning squids significantly increased the relative amounts of 16:0 and 20:4n6, both which have been used as indicators of carnivores and top predators respectively [40, 41]. The results might expect that maturing squids primarily feed on herbivores, and mature and spawning ones switch to feed on carnivorous prey items. It is noted that the mature squids may prey on similar prey items, evidenced by the overlaps in the isotopic niche (Fig. 3d) and FA profiles (Fig. 4c). Comparing to small prey species, the one at higher trophic position has greater energy density [20, 21], and would improve the metabolic energy of consumers [22]. However, prey species at higher trophic position may perform better to avoid predation [23]. Consumers must trade-off energy acquisition and the cost of capturing prey [6]. Therefore, the squid may balance energy expenditure and gain to maximize energy intake through ontogenetic shift in diet, coupled with broadening prey spectrum.

The strategy of energy acquisition for reproduction is independent from body size that has been considered as an important factor linked to feeding ability [15, 25]. LMMs results indicated that reproductive energy was not correlated with mantle length (Additional file 1: Tables S2, S3). This seems contrast to previous reports that larger individuals invest much more reproductive energy [14], and many species do exhibit reproductive energy output scaling with body size [51]. However, most of the studies linearized reproductive investment to body size without considering the potential effects of maturity state. In squid species, notably, somatic growth is gradually decreasing after reaching maturation [10], while the reproductive system expands dramatically, especially during physiologically maturing stage [12]. Thus, the influence of body size on reproductive energy acquisition may become less important after the onset of maturation in squids.

Resource availability is also an important factor that influences consumer’s feeding habits [52, 53]. For squid species, many of them have been reported that feeding behavior is related to the availability of food resources [17, 53], potentially linking to their opportunistic life-history and voracious foraging behavior [10]. In the present study, we observed significant variations in the weekly surface chlorophyll-*a* concentration in the sampling area (Additional file 1: Fig. S1), suggesting that the primary production are temporal variable [54]. Unexpectedly, the reproductive energy was not related to the surface chlorophyll-*a* concentration (Additional file 1: Tables S2, S3). The southwestern Atlantic system has high biological production because of the high variability of physio-chemical and biological attributes occurring at the shelf and slope water columns [55]. Therefore, the prey community can be resilient to the temporal change of productivity and maintain a status of dynamic equilibrium [56]. Indeed, the sampling stations were apparently not situated in water columns with high chlorophyll-*a* concentration (Additional file 1: Fig. S3a). This case can indirectly provide evidence to explain the non-significant relationship between reproductive energy and chlorophyll-*a* concentration.

## Conclusion

The semelparous Argentinean shortfin squid invests large amounts of energy for reproduction during maturation. In order to meet the energy demand, the squid ontogenetically shifts feeding habits from lower to higher trophic prey items during maturation. However, the shift is not equal to a complete trophic level. Instead, the squid broadens the prey spectrum, coupled with increasing intake of higher trophic prey. Energy acquisition for reproduction was independent of body size and primary production. This strategy of energy acquisition for reproduction may be evolutionarily selected by the squid to maximize reproductive values by balancing energy intake and expenditure from foraging. To our best knowledge, this work is the first study that uses multiple techniques including energy density, stable isotopes and fatty acid biomarkers to investigate energy acquisition strategy among semelparous species. The results put forward our understanding of the squid’s life-history in terms of energy acquisition for reproduction. Future studies should aim to confirm whether the strategy identified in this study holds for other semelparous species, which have common monocyclic life-histories but can vary in breeding patterns and therefore, reproductive strategies.

## Methods

### Sampling region

The squid specimens were collected over the Patagonian shelf of the southwest Atlantic Ocean (Fig. 1). It is well known that the southwest Atlantic is characterized by the presence of the Brazil Current (BC), a southward-flowing warm western boundary current, and the northward-flowing Malvinas Current (MC), in which both currents encounter at approximately 38°S [57]. Meanwhile, intense frontal transitions at various near shore locations and along the shelf break promote vertical circulations that inject nutrients into the upper layer, leading to the enhanced growth of phytoplankton [55]. Consequently, the waters along and across the shelf and slope are inhabited by diverse organisms, including mammal, bird, fish and cephalopod species in all life stages [56].

### Specimens and process

Squid specimens were obtained from the catches of commercial jigging fishery that operated in the high seas of southwest Atlantic (61° 09′ W ~ 62° 53′ W, 46° 08′ S ~ 47° 51′ S; Fig. 1) from January 8th to March 4th in 2019. Specimens were randomly collected onboard, and frozen immediately at – 30 °C for laboratory experiments. After defrosted at room temperature in laboratory, each specimen was taken dorsal mantle length (ML) to the nearest 1.0 mm and body weight (BW) to the nearest 1.0 g. Each specimen was dissected to identify sex and assigned a macro-scale maturity stage. The macro-scale maturity stage was after the scales proposed by ICES [37] and Lin et al. [38]: I immature, II developing, III physiologically maturing, IV–V, physiologically mature, VI, functionally mature, VII, spawning, VIII, spent. 152 specimens were sexed and assigned the maturity stages. Preliminary analysis indicated that about one fifth of the specimens were at stages I–II, another one fifth at stage III, and the remaining at stages IV–VII. No spent individuals were found, probably because spent individuals die shortly after spawning [58].

Since the squid develops reproductive system from stage III [10], we randomly selected 68 female specimens at maturity stages from III to VII for energy density analysis, stable isotope analysis and fatty acids analyses (12 at stage III, 15 at stage IV, 15 at stage V, 15 at stage VI, and 11 at stage VII). For each selected specimen, the ovary, nidamental glands and eggs were taken and lyophilized to constant weight in a freeze-dried chamber (Scientz-10 N lab lyophilizer, Ningbo Scientz Biotechnology Co., LTD.). The dry weight of each tissue was weighed to the nearest 0.1 mg, and then ground into fine powder using Scientz-48 grinder (Ningbo Scientz Biotechnology Co., LTD.).

### Reproductive energy

An approximately 1.5–4.0 g of powdered tissue was used to determine the energy density (kJ g^−1^) of the ovary, nidamental glands and eggs of each squid specimen, separately, using an automatic isoperibol calorimeter (Model 6400, Parr Instrument Company, Moline, IL, USA). The powdered tissue was gently added to a capsule and then placed into the capsule holder of the calorimeter, which allows for automatically determining the energy density within several minutes.

The absolute energy (kJ) of a given tissue was calculated as the energy density multiplied by the dry tissue weight. Then, the reproductive energy was estimated as the sum of absolute energy of the ovary, nidamental glands and eggs.

### Fatty acids analyses

An approximately 1 g of powdered ovary was used to extract lipids with a methanol: dichloromethane: water solvent mixture (20:10:8 by volume) according to Bligh and Dyer method [59]. The extracted lipids were stored at − 20 °C in methanol: dichloromethane (2:1 by volume) with 0.01% butylated hydroxytoluene (BHT) as antioxidant for fatty acid methyl esters (FAME) analysis within 24–48 h. The lipid extracted tissue was recycled and lyophilized to constant weight for stable isotope analysis.

A modification of GAQSIQ method [60] was used to analyze the FAME of the squid’s ovary. The method has been successfully tested in soma, ovary and digestive gland samples of *I. argentinus* [35] and *Dosidicus gigas* [61]. FAME were determined using an Agilent 7890B Gas Chromatography (GC) coupled to a 5977A series Mass Spectrometer Detector (MSD, Agilent Technologies, Inc. USA), equipped with a fused silica 60 m × 0.25 nm open tubular column (HB-88: 0.20 μm, Agilent Technologies, Inc. USA). Individual FAME was identified by the retention times and mass spectra with a known concentration internal standard 19:0 (GLC 37, Nu-Chek Prep, Inc.). The separation was carried out with helium as the carrier gas, and a thermal gradient programed from 125 to 250 °C, with the auxiliary heater at 280 °C.

Total content of fatty acids (total FAs) was determined as milligram per gram of dry mass (mg/g dry mass), based on the mass fraction of FAMEs relative to the internal standard 19:0 [60]. Individual fatty acid (FA) was expressed as percentage of total FAs. Individual fatty acid that accounted for less than 0.5% of the total FAs was excluded from statistical analyses below. The individual fatty acid was also grouped into saturated fatty acids (SFA), monounsaturated fatty acids (MUFA), and polyunsaturated fatty acids (PUFA), which were also expressed as percentage of total FAs.

### Stable isotope analysis

Lipid fraction in soft tissues tends to be depleted in ^13^C isotope, and lipid extraction has become a standard procedure in stable isotope analysis [62, 63]. The lipid-extracted ovary samples (see “Fatty acids analysis”) were used to determined carbon (δ^13^C) and nitrogen (δ^15^N) stable isotope values. A powdered sub-sample of ovary tissue (≈ 0.3 mg) was placed into tin capsule and analyzed in a SerCon Integra 2 integrated elemental analyzer and an isotope ratio mass spectrometer (EA-IRMS) at the Stable Isotope Core Laboratory in Third Institute of Oceanography (Ministry of Natural Resources, China). Isotopic values are reported in standard δ-notion in parts per thousand (‰), where δ^13^C or δ^15^N = [(R_sample_/R_standard_) − 1] × 1000, with R_sample_ and R_standard_ representing the ratios of ^13^C/^12^C and ^15^ N/^14^ N of the sample and the standard reference material, respectively. The reference material was Vienna Pee Dee Belemnite (VPDB) for carbon and atmospheric nitrogen (N_2_) for nitrogen. The measurement errors were approximately 0.02‰ and 0.02‰ for δ^13^C and δ^15^N, respectively.

### Statistical analyses

Significant differences (*p* < 0.05) among maturity stages were tested for the tissue energy density, reproductive energy, stable isotopes, total fatty acids content and relative amount of individual fatty acid. All data were checked for normality of distribution with the one-sample Kolmogorov–Smirnov test and for homogeneity of the variances with the Levene’s test [64]. When the normality was satisfied, one-way analyses of variance (ANOVA) was applied to determine the significant difference between maturity stages, followed by Tukey’s honestly significant post-hoc test. When normal distribution and/or homoscedasticity were not achieved, data were subjected to a Kruskall–Wallis nonparametric test and a Games–Howell post hoc test was performed [64].

To determine the energy acquisition strategy during maturation, we evaluated the ovary isotopic niche based on Bayesian inference, and assessed the similarity of ovary fatty acid profiles. The ovary isotopic niche can reflect the dietary niche characteristics of the squid [32], and the ovary fatty acid profiles can be used to evaluate the potential similarity of dietary items [34]. We calculated the isotopic niche widths for each maturity stage using Stable Isotope Bayesian Ellipses in R (SIBER, [39]), including the Bayesian estimate of standard ellipse area (SEA_b_), the corrected standard ellipse area (SEA_c_) and the isotopic niche overlap estimated as the ratio of overlap proportion and the sum of non-overlap areas between each consecutive SEAc (non-overlap SEA_c_ proportion) based on 1000 replications.

We applied analysis of similarity (ANOSIM) to examine differences in the overall fatty acid profiles between maturity stages. In addition, non-metric multidimensional scaling (NMDS) was applied to visualize the differences in the overall FA profiles. As individual FA was expressed as percentage of total FAs, a square-rooted transformation was used to avoid over-emphasis of extreme values [34]. A Bray–Curtis dissimilarity matrices was employed in the ANOSIM and NMDS [65]. The analyses were performed in the package ‘vegan’ in R platform [66].

We further applied linear mixed-effects models (LMMs) [36] to investigate the energy acquisition strategy for reproduction in *I. argentinus*. LMMs were respectively performed the reproductive energy on δ^15^N values and selected fatty acid profiles, using maturity stage to account for sexual development effects and unexplained differences among individuals from different maturity stages. The selected FA profiles were those FAs that were found significant differences between maturity stages. Log-transformed mantle length of the squid and sea surface chlorophyll-*a* concentration (Chla) were also considered as explanatory variables to account for the effects of body size and primary production, respectively. The Chla data were downloaded from NOAA (https://oceanwatch.pifsc.noaa.gov/erddap/). To reduce the bias of daily variation, we used weekly Chla (Dataset ID: noaa_snpp_chla_weekly). LMMs were performed in the package ‘lme4’ in R platform [66].

## Supplementary Information

**Additional file 1: Table S1.** Fatty acid composition of ovary of Argentinean shortfin squid. **Table S2.** Linear mixed-effects models results for reproductive energy relation to nitrogen stable isotope ratios, mantle length and chlorophyll-a concentration. **Table S3.** Linear mixed-effects models results for reproductive energy relation to selected fatty acids, mantle length and chlorophyll-a concentration. **Fig. S1.** Mantle length and body weight of Argentinean shortfin squid for this work. **Fig. S2.** Body size distribution of Argentinean shortfin squid with sexual maturation. **Fig. S3.** Sea surface chlorophyll-a (Chla, mg g^-1^) concentration around the sampling stations in the Southwest Atlantic Ocean.

## Data Availability

All data that support the findings of this study are included in the manuscript and supplementary material. Data are however used under license from the Distant Squid Fisheries Sci-Tech Group (SHOU), and the usage is only permitted from the authors and Distant Squid Fisheries Sci-Tech Group (SHOU) upon reasonable request.
