# Supplementary material for: Energy acquisition strategy for reproduction in a semelparous squid
Source: Front Zool. 2022 Nov 16;19:28. doi: 10.1186/s12983-022-00473-w (PMC9670511; doi:10.1186/s12983-022-00473-w)
Supplement: Supplementary file 1 — Additional file 1: Table S1. Fatty acid composition of ovary of Argentinean shortfin squid. Table S2. Linear mixed-effects models results for reproductive energy relation to nitrogen stable isotope ratios, mantle length and chlorophyll-a concentration. Table S3. Linear mixed-effects models results for reproductive energy relation to selected fatty acids, mantle length and chlorophyll-a concentration. Fig. S1. Mantle length and body weight of Argentinean shortfin squid for this work. Fig. S2. Body size distribution of Argentinean shortfin squid with sexual maturation. Fig. S3. Sea surface chlorophyll-a (Chla, mg g-1) concentration around the sampling stations in the Southwest Atlantic Ocean. [file 12983_2022_473_MOESM1_ESM.docx]

**Supplementary materials**

**Energy acquisition strategy for reproduction in a semelparous squid**

Dongming Lin^1,2,3,4*^, Na Zang^1^, Kai Zhu^5^, Gang Li^1,2,3,4*^, Xinjun Chen^1,2,3,4*^

^1^ College of Marine Science, Shanghai Ocean University, Shanghai 201306, China

^2^ Key Laboratory of Sustainable Exploitation of Oceanic Fisheries Resources, Ministry of Education, Shanghai 201306, China

^3^ National Engineering Research Center for Oceanic Fisheries, Ministry of Science and Technology, Shanghai 201306, China

^4^ Key Laboratory of Oceanic Fisheries Exploration, Ministry of Agriculture and Rural Affairs, Shanghai 201306, China

^5^ Zhejiang Marine Fisheries Research Institute, Zhoushan 316021, China

^*^Corresponding author:

Dongming Lin, email: [dmlin@shou.edu.cn](mailto:dmlin@shou.edu.cn);

Gang Li, email: [g-li@shou.edu.cn](mailto:g-li@shou.edu.cn);

Xinjun Chen, email: xjchen@shou.edu.cn.

**Supplementary tables**

**Table S1 Fatty acid composition of ovary of Argentinean shortfin squid.** Values are mean ± SD; different superscript letters within rows represent significant differences determined by post-hoc test. SFA saturated fatty acids, MUFA monounsaturated fatty acids, PUFA polyunsaturated fatty acids, total FAs total content of fatty acids. total FAs is reported as dry tissue weight (mg/g dry mass), other values are reported as percentages of total FAs (% total FAs). FA<0.5% include 15:0, 20:0, 21:0, 22:0, 23:0, 24:0, 14:1n5, 15:1n5, 17:1n7, 22:1n9, 24:1n9, 18:3n6, 18:3n3, 20:3n6.

| Fatty_acid | III | IV | V | VI | VII |
| --- | --- | --- | --- | --- | --- |
| 14:0 | 1.50±0.15^c^ | 1.86±0.25^b^ | 2.21±0.16^a^ | 2.15±0.22^a^ | 2.07±0.26^ab^ |
| 16:0 | 21.48±2.58^b^ | 24.90±2.51^a^ | 25.71±1.52^a^ | 26.37±0.62^a^ | 24.19±1.97^a^ |
| 16:1n7 | 0.65±0.16 | 0.77±0.19 | 0.62±0.19 | 0.77±0.21 | 0.70±0.16 |
| 17:0 | 0.75±0.25 | 0.77±0.17 | 0.74±0.24 | 0.74±0.09 | 0.86±0.12 |
| 18:0 | 8.06±0.83^a^ | 7.37±1.28^ab^ | 6.98±0.33^b^ | 6.82±0.57^b^ | 7.21±0.52^ab^ |
| 18:1n9 | 10.42±1.88^a^ | 9.69±1.56^ab^ | 8.54±1.94^b^ | 9.23±0.96^ab^ | 8.26±0.81^b^ |
| 18:2n6 | 0.64±0.40 | 0.56±0.44 | 0.47±0.06 | 0.43±0.04 | 0.53±0.11 |
| 20:1 | 9.59±0.57^a^ | 8.50±0.76^b^ | 8.03±0.55^bc^ | 7.77±0.36^c^ | 8.32±0.37^bc^ |
| 20:2 | 0.58±0.17^b^ | 0.67±0.21^ab^ | 0.75±0.14^ab^ | 0.65±0.11^ab^ | 0.84±0.13^a^ |
| 20:3n3 | 1.09±0.27^b^ | 1.16±0.31^ab^ | 1.25±0.21^ab^ | 1.19±0.24^ab^ | 1.46±0.25^a^ |
| 20:4n6 | 1.05±0.34^b^ | 1.22±0.32^ab^ | 1.29±0.25^ab^ | 1.30±0.29^ab^ | 1.59±0.30^a^ |
| 22:2n6 | 3.02±0.48 | 2.87±0.30 | 3.07±0.16 | 2.95±0.10 | 3.09±0.13 |
| 20:5n3 | 10.46±1.26^b^ | 10.04±0.59^b^ | 10.74±0.39^ab^ | 10.61±0.49^b^ | 10.93±0.50^a^ |
| 22:6n3 | 26.97±1.20 | 26.54±0.79 | 27.04±1.33 | 26.56±0.71 | 26.96±1.33 |
| ∑FA<0.5% | 3.73±2.09 | 3.09±2.11 | 2.55±0.19 | 2.45±0.20 | 2.98±0.55 |
| ∑SFA | 33.39±2.17^b^ | 36.26±1.69^a^ | 36.73±1.39^a^ | 37.10±0.61^a^ | 35.59±1.89^a^ |
| ∑MUFA | 11.33±0.99^a^ | 10.17±0.92^b^ | 9.40±0.49^b^ | 9.30±0.56^b^ | 9.92±0.41b |
| ∑PUFA | 44.60±2.32^ab^ | 43.66±2.36^b^ | 45.15±1.36^ab^ | 44.19±0.84^ab^ | 46.03±1.77^a^ |
| total FAs | 108.68±18.44^b^ | 136.88±18.22^a^ | 138.66±7.08^a^ | 141.95±8.14^a^ | 130.58±6.51^a^ |

**Table S2 Linear mixed-effects models results for reproductive energy relation to nitrogen stable isotope ratios, mantle length and chlorophyll-*a* concentration.** In the model analysis, mantle length was log-transformed.

| ***Random effects:*** | |  |  |  |
| --- | --- | --- | --- | --- |
| Groups | Name | Variance | Std.Dev. |  |
| Maturity stage | (Intercept) | 0.27 | 0.52 |  |
| Residual |  | 0.12 | 0.35 |  |
| ***Fixed effects:*** | |  |  |  |
|  | Estimate | Std. Error | t value | Pr(>\|t\|) |
| (Intercept) | -5.03 | 4.60 | -1.09 | 0.28 |
| δ^15^N | 0.24 | 0.08 | 3.02 | 0.0038 |
| Mantle length | 1.25 | 0.76 | 1.65 | 0.10 |
| Chlorophyll- *a* concentration | -0.03 | 0.13 | -0.20 | 0.84 |

**Table S3 Linear mixed-effects models results for reproductive energy relation to selected fatty acids, mantle length and chlorophyll-*a* concentration**. Selected fatty acids were those significant differences in relative amounts between maturity stages, see Table S1; mantle length was log-transformed in the model analysis.

| ***Random effects:*** | |  |  |  |
| --- | --- | --- | --- | --- |
| Groups | Name | Variance | Std.Dev. |  |
| Maturity stage | (Intercept) | 0.15 | 0.39 |  |
| Residual |  | 0.09 | 0.30 |  |
| ***Fixed effects:*** | |  |  |  |
|  | Estimate | Std. Error | t value | Pr(>\|t\|) |
| (Intercept) | -8.78 | 6.53 | -1.34 | 0.19 |
| C14.0 | 2.03 | 0.68 | 2.97 | 0.00 |
| C16.0 | 0.55 | 0.33 | 1.68 | 0.10 |
| C18.0 | 0.89 | 0.42 | 2.13 | 0.04 |
| C18.1n9 | 0.18 | 0.22 | 0.83 | 0.41 |
| C20.1 | -0.82 | 0.62 | -1.31 | 0.20 |
| C20.2 | -0.89 | 0.81 | -1.09 | 0.28 |
| C20.5n3 | 0.24 | 0.70 | 0.34 | 0.74 |
| C20.4n6 | 1.25 | 0.48 | 2.60 | 0.01 |
| Chlorophyll- *a* concentration | -0.10 | 0.16 | -0.61 | 0.55 |
| Mantle length | 1.21 | 0.72 | 1.69 | 0.10 |

**Supplementary figures**


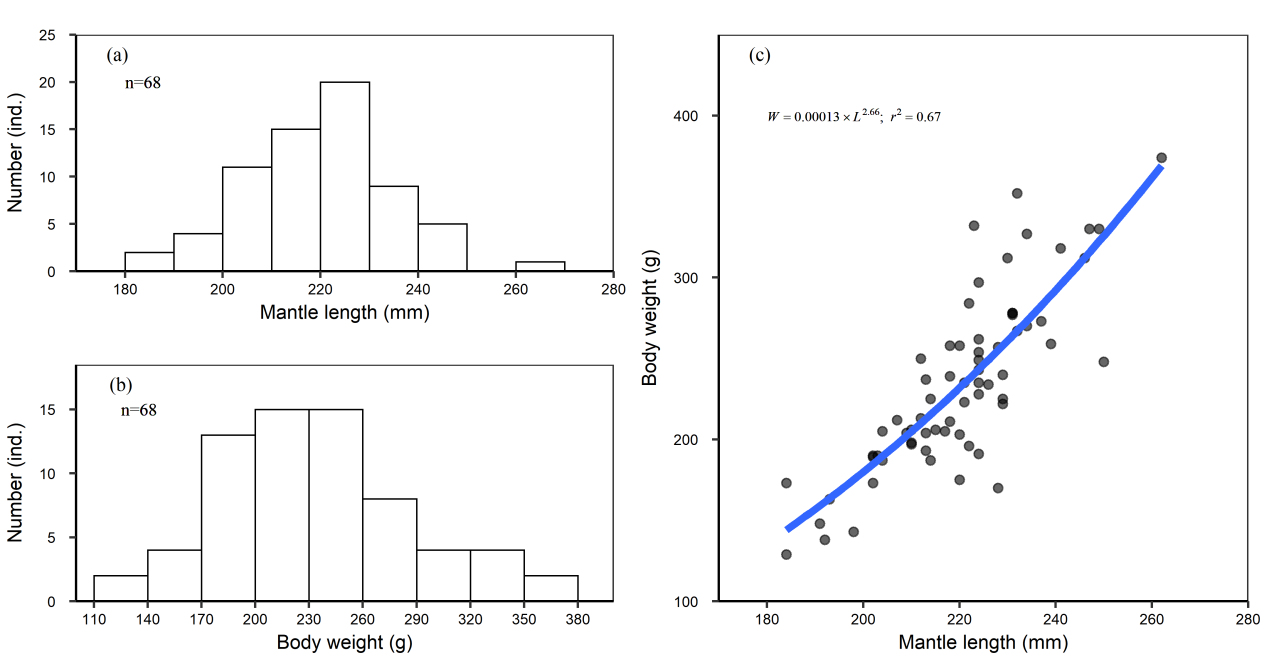


**Figure S1 Mantle length and body weight of Argentinean shortfin squid for this work**. (a), Mantle length distribution; (b), Body weight distribution; (c), Relationship between mantle length and body weight.


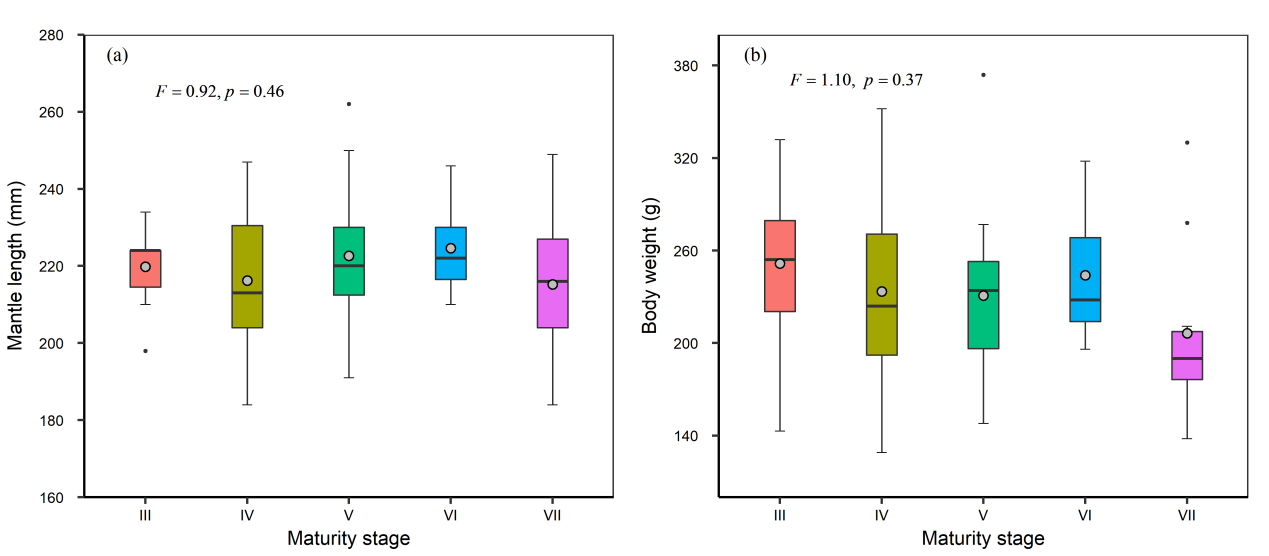


**Figure S2 Body size distribution of Argentinean shortfin squid with sexual maturation**. (a), Mantle length; (b), Body weight. The boxplot horizontal line and grey solid point respectively denote the median and mean, while upper and lower hinges respectively represent the 25th and 75^th^ percentiles.


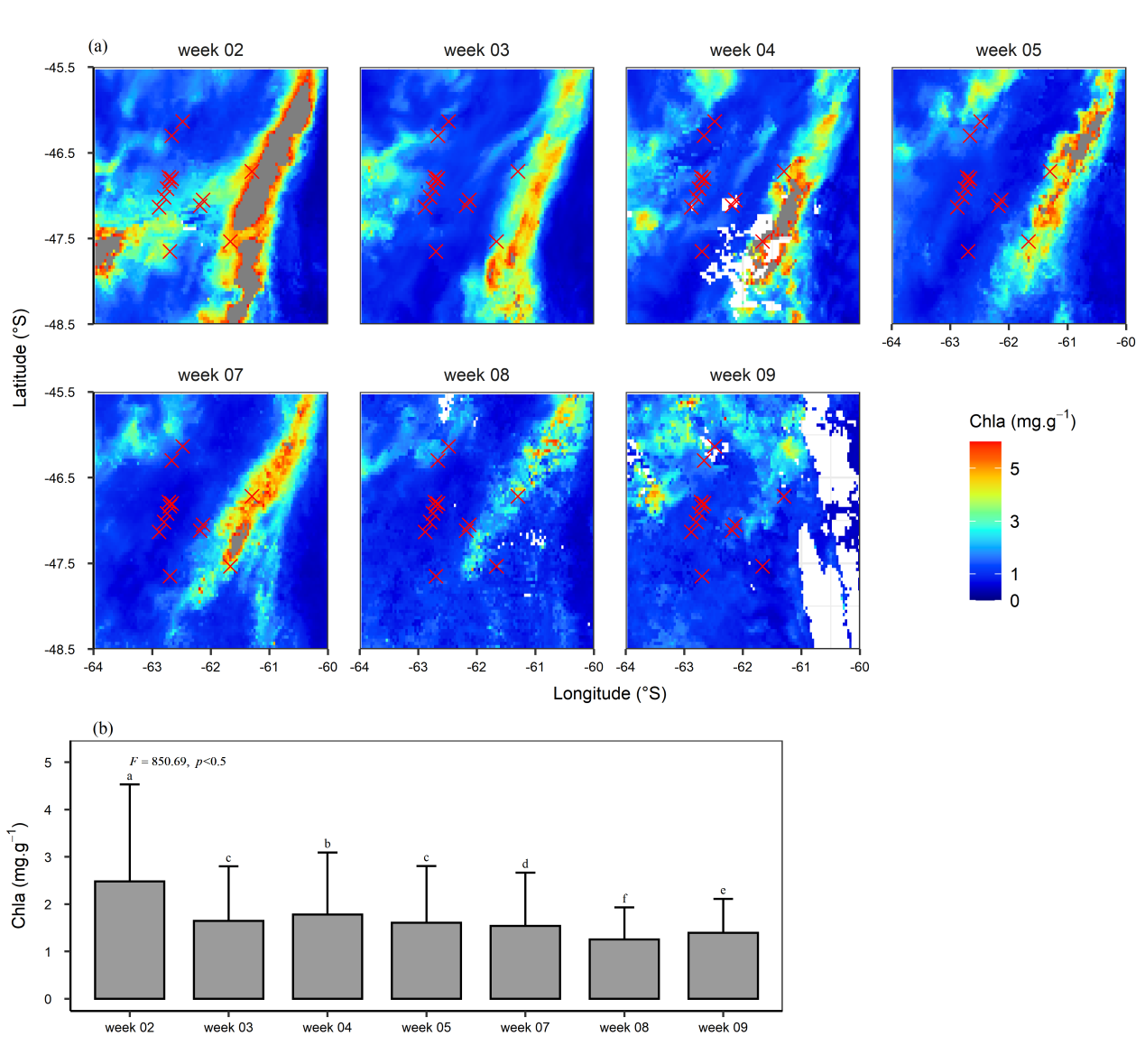


**Figure S3 Sea surface chlorophyll-*a* (Chla, mg.g^-1^) concentration around the sampling stations in the Southwest Atlantic Ocean**. (a) Raster plot of weekly Chla that downloaded from NOAA (<https://oceanwatch.pifsc.noaa.gov/erddap/griddap/>) with the Dataset ID ‘noaa_snpp_chla_weekly’; (b) Mean values of weekly Chla. Red cross (x) presents the sampling station; week02, week03, …, and week09 represent the week indices of 2019; bars in (b) represent the standard deviance. Different letters above the bars indicated significant difference at statistical level 0.05.

**Datasets that were used in the manuscript**

**1. Dataset of reproductive tissue energy density**

| maturity stage | Ovary energy density | Enegy density of Nidamental glands | Energy density of oviducts |
| --- | --- | --- | --- |
| III | 20.9716 | 20.3167 |  |
| III | 21.0239 | 20.4389 |  |
| III | 21.5594 | 20.7161 |  |
| III | 21.7169 | 20.1624 |  |
| III | 21.9601 | 20.1938 |  |
| III | 22.1923 | 20.3946 |  |
| III | 22.6705 | 19.7356 |  |
| III | 22.8554 | 20.5340 |  |
| III | 22.9777 | 20.0648 |  |
| III | 23.2557 | 19.9930 |  |
| III | 23.4683 | 20.5616 |  |
| III | 23.5166 | 19.3558 |  |
| IV | 21.3429 | 20.8799 | 24.7841 |
| IV | 23.1621 | 20.1129 | - |
| IV | 23.3517 | 18.7719 | 25.7088 |
| IV | 23.5112 | 18.8537 | 25.2983 |
| IV | 23.5949 | 19.6431 | 25.6323 |
| IV | 23.8339 | 19.9238 | 24.8539 |
| IV | 23.8830 | 20.4453 | 24.7989 |
| IV | 23.8963 | 19.4865 | - |
| IV | 23.9747 | 18.8758 | 24.4370 |
| IV | 24.1399 | 20.1440 | 25.4296 |
| IV | 24.1936 | 20.1123 | 24.6975 |
| IV | 24.2087 | 19.9125 | - |
| IV | 24.2305 | 19.2292 | 25.7234 |
| IV | 24.3138 | 20.3756 | 26.4633 |
| IV | 24.7405 | 20.0238 | - |
| V | 22.8919 | 19.4811 | 25.0791 |
| V | 22.9585 | 20.5622 | 25.4070 |
| V | 23.1605 | 20.1378 | 25.2052 |
| V | 23.2141 | 19.7769 | 25.3814 |
| V | 23.7639 | 19.8924 | 25.1042 |
| V | 23.1225 | 19.5532 | 25.5849 |
| V | 23.4841 | 18.6371 | 24.6978 |
| V | 23.8902 | 20.0438 | 25.5253 |
| V | 23.9966 | 18.9182 | 25.5577 |
| V | 24.0629 | 19.7649 | 25.5896 |
| V | 24.3130 | 20.2790 | 25.7865 |
| V | 24.1411 | 19.4395 | 25.1747 |
| V | 24.3744 | 19.7101 | 25.3691 |
| V | 24.0793 | 20.3890 | 25.3686 |
| V | 24.3862 | 19.7670 | 25.7545 |
| VI | 22.9008 | 19.9211 | 25.8434 |
| VI | 24.2827 | 20.0148 | 25.4879 |
| VI | 23.2453 | 19.6651 | 25.3758 |
| VI | 23.3977 | 20.2490 | 25.6647 |
| VI | 23.7161 | 19.9382 | 24.7470 |
| VI | 24.8080 | 20.1926 | 25.7839 |
| VI | 23.8908 | 19.8389 | 25.2196 |
| VI | 23.9517 | 19.5237 | 25.8629 |
| VI | 23.9895 | 19.6929 | 25.7648 |
| VI | 24.0900 | 19.8144 | 25.5399 |
| VI | 24.1713 | 20.1392 | 25.3727 |
| VI | 24.3138 | 19.7974 | 25.7300 |
| VI | 24.3438 | 19.9436 | 25.6591 |
| VI | 24.3572 | 20.0550 | 25.4839 |
| VI | 24.4559 | 20.5150 | 25.7014 |
| VII | 22.2872 | 19.2943 | 24.9433 |
| VII | 22.5901 | 19.8159 | 25.7300 |
| VII | 22.6196 | 20.1999 | 25.0391 |
| VII | 22.7608 | 20.0828 | 25.7759 |
| VII | 22.7813 | 20.4527 | 25.1283 |
| VII | 22.9036 | 20.5016 | 24.6873 |
| VII | 22.9356 | 20.0861 | 24.9542 |
| VII | 23.1531 | 20.3369 | 25.5923 |
| VII | 23.9682 | 19.6526 | 25.6971 |
| VII | 24.3077 | 20.2525 | 24.9655 |

**2. Dataset of stable isotopes**

| Maturity stage | Mantle length (mm) | Nitrogen stable isotope ratios | Corbon stable isotope ratios |
| --- | --- | --- | --- |
| III | 224 | 13.03 | -17.83 |
| III | 234 | 13.55 | -16.99 |
| III | 224 | 13.62 | -17.21 |
| III | 223 | 13.79 | -16.97 |
| III | 217 | 13.81 | -17.23 |
| III | 224 | 14.07 | -17.80 |
| III | 212 | 14.21 | -17.63 |
| III | 204 | 13.30 | -16.75 |
| III | 228 | 14.33 | -17.15 |
| III | 210 | 13.56 | -17.83 |
| IV | 247 | 14.79 | -17.44 |
| IV | 232 | 13.92 | -17.94 |
| IV | 234 | 13.71 | -18.02 |
| IV | 204 | 13.82 | -16.70 |
| IV | 224 | 13.95 | -17.39 |
| IV | 221 | 13.99 | -17.64 |
| IV | 204 | 14.52 | -17.95 |
| IV | 213 | 14.61 | -17.62 |
| IV | 213 | 14.64 | -17.47 |
| IV | 230 | 14.82 | -17.29 |
| IV | 237 | 14.91 | -17.89 |
| IV | 202 | 15.18 | -16.58 |
| IV | 209 | 15.38 | -16.82 |
| IV | 212 | 15.47 | -17.09 |
| IV | 184 | 15.87 | -15.90 |
| V | 226 | 13.96 | -17.60 |
| V | 220 | 14.02 | -17.49 |
| V | 229 | 14.17 | -17.72 |
| V | 220 | 14.46 | -18.03 |
| V | 218 | 14.57 | -17.50 |
| V | 222 | 14.66 | -17.40 |
| V | 202 | 14.70 | -16.24 |
| V | 232 | 14.73 | -17.14 |
| V | 218 | 14.99 | -17.93 |
| V | 229 | 15.14 | -17.12 |
| V | 220 | 15.23 | -16.41 |
| V | 262 | 15.46 | -17.90 |
| V | 213 | 15.49 | -17.10 |
| V | 221 | 15.76 | -15.82 |
| V | 191 | 15.88 | -16.13 |
| VI | 214 | 14.94 | -17.74 |
| VI | 250 | 14.03 | -17.47 |
| VI | 241 | 14.26 | -17.97 |
| VI | 203 | 14.39 | -17.56 |
| VI | 224 | 14.64 | -17.85 |
| VI | 231 | 14.67 | -17.70 |
| VI | 239 | 14.75 | -17.54 |
| VI | 215 | 14.98 | -17.91 |
| VI | 246 | 14.99 | -17.99 |
| VI | 207 | 15.04 | -16.28 |
| VI | 231 | 15.21 | -17.05 |
| VI | 229 | 15.48 | -17.73 |
| VI | 224 | 15.76 | -17.71 |
| VI | 210 | 15.90 | -15.71 |
| VI | 222 | 16.64 | -16.62 |
| VII | 220 | 13.50 | -17.39 |
| VII | 228 | 13.95 | -17.12 |
| VII | 228 | 14.60 | -17.00 |
| VII | 224 | 15.03 | -16.94 |
| VII | 218 | 15.06 | -17.45 |
| VII | 224 | 15.14 | -17.09 |
| VII | 214 | 15.34 | -15.93 |
| VII | 184 | 15.42 | -17.21 |
| VII | 202 | 15.54 | -17.07 |
| VII | 231 | 15.70 | -17.08 |

**3. Dataset of fatty acid profiles.** SFA saturated fatty acids, MUFA monounsaturated fatty acids, PUFA polyunsaturated fatty acids, total FAs total content of fatty acids. total FAs is reported as dry tissue weight (mg/g dry weight), other values are reported as percentages of total FAs (% total FAs).

| Maturity stage | 14:0 | 16:0 | 16:1n7 | 17:0 | 18:0 | 18:1n9t | 18:1n9c | 20:1 | 20:2 | 20:3n3 | 20:4n6 | 22:2n6 | 20:5n3 | 22:6n3 | SFA | MUFA | PUFA | Total FAs |
| --- | --- | --- | --- | --- | --- | --- | --- | --- | --- | --- | --- | --- | --- | --- | --- | --- | --- | --- |
| III | 1.29 | 20.90 | 0.59 | 0.78 | 8.37 | 0.36 | 10.38 | 9.90 | 0.40 | 1.12 | 1.23 | 2.94 | 10.39 | 27.60 | 33.12 | 22.30 | 44.59 | 113.46 |
| III | 1.61 | 22.98 | 0.67 | 0.69 | 7.26 | 0.33 | 10.77 | 9.57 | 0.55 | 1.07 | 1.14 | 2.72 | 9.71 | 26.93 | 34.45 | 22.43 | 43.12 | 117.15 |
| III | 1.28 | 21.29 | 0.56 | 0.81 | 9.04 | 0.38 | 11.34 | 9.27 | 0.55 | 1.10 | 1.18 | 2.84 | 9.89 | 26.00 | 34.57 | 22.78 | 42.65 | 128.29 |
| III | 1.55 | 25.21 | 0.54 | 0.75 | 8.74 | 0.61 | 8.91 | 8.85 | 0.62 | 0.91 | 0.98 | 2.58 | 9.06 | 27.01 | 38.01 | 19.89 | 42.10 | 133.56 |
| III | 1.33 | 19.55 | 0.49 | 0.78 | 8.03 | 0.21 | 10.76 | 10.17 | 0.57 | 0.83 | 0.52 | 3.14 | 10.84 | 27.68 | 32.16 | 22.95 | 44.89 | 94.29 |
| III | 1.67 | 20.90 | 0.69 | 0.20 | 8.69 | 0.36 | 11.43 | 9.60 | 0.62 | 1.01 | 0.68 | 2.91 | 10.30 | 26.20 | 33.79 | 23.15 | 43.06 | 94.04 |
| III | 1.45 | 22.70 | 0.61 | 0.69 | 8.31 | 0.63 | 10.94 | 9.03 | 0.62 | 1.13 | 1.23 | 2.61 | 9.07 | 27.05 | 34.99 | 22.30 | 42.72 | 127.83 |
| III | 1.69 | 14.57 | 1.05 | 1.23 | 6.04 | 0.67 | 4.56 | 10.24 | 0.99 | 1.71 | 1.56 | 4.22 | 13.33 | 23.44 | 31.27 | 19.52 | 49.21 | 71.47 |
| III | 1.47 | 21.97 | 0.52 | 0.79 | 7.93 | 0.32 | 10.49 | 9.09 | 0.52 | 0.90 | 0.91 | 3.05 | 10.77 | 26.06 | 34.70 | 21.75 | 43.55 | 113.73 |
| III | 1.54 | 21.23 | 0.63 | 0.79 | 7.25 | 0.33 | 10.74 | 9.83 | 0.52 | 1.29 | 1.37 | 2.94 | 10.19 | 26.86 | 32.99 | 22.64 | 44.37 | 105.56 |
| III | 1.40 | 22.61 | 0.74 | 0.63 | 8.09 | 0.39 | 8.58 | 8.75 | 0.40 | 0.75 | 0.56 | 2.88 | 10.21 | 28.65 | 35.36 | 19.85 | 44.79 | 108.21 |
| IV | 1.68 | 17.99 | 0.94 | 1.14 | 6.07 | 0.86 | 4.88 | 8.13 | 1.08 | 1.78 | 1.66 | 3.51 | 10.46 | 26.03 | 34.43 | 17.84 | 47.72 | 98.53 |
| IV | 1.50 | 24.30 | 0.86 | 0.55 | 9.53 | 0.54 | 9.61 | 9.33 | 0.37 | 0.77 | 0.80 | 2.61 | 9.35 | 26.44 | 37.61 | 21.19 | 41.19 | 132.59 |
| IV | 1.98 | 25.99 | 0.73 | 0.70 | 7.94 | 0.86 | 9.44 | 8.74 | 0.80 | 0.93 | 1.03 | 2.64 | 9.33 | 25.61 | 38.12 | 20.68 | 41.20 | 145.77 |
| IV | 2.14 | 26.16 | 0.75 | 0.74 | 6.76 | 0.73 | 9.81 | 7.71 | 0.76 | 1.27 | 1.42 | 2.75 | 9.75 | 25.53 | 37.51 | 20.01 | 42.48 | 148.11 |
| IV | 1.74 | 25.38 | 0.61 | 0.77 | 7.29 | 0.79 | 7.83 | 7.93 | 0.78 | 1.36 | 1.52 | 2.78 | 9.83 | 27.84 | 36.88 | 18.07 | 45.06 | 126.10 |
| IV | 1.54 | 22.70 | 0.62 | 0.80 | 8.69 | 0.38 | 11.14 | 10.06 | 0.55 | 1.34 | 1.43 | 2.60 | 9.41 | 25.23 | 35.38 | 23.12 | 41.50 | 142.06 |
| IV | 1.65 | 25.58 | 1.02 | 0.67 | 7.75 | 0.89 | 8.68 | 7.82 | 0.51 | 0.96 | 0.78 | 3.00 | 10.91 | 26.55 | 37.16 | 19.28 | 43.56 | 162.18 |
| IV | 2.11 | 25.86 | 0.51 | 0.73 | 6.69 | 0.71 | 9.53 | 8.48 | 0.55 | 1.04 | 1.14 | 2.77 | 9.73 | 26.65 | 37.09 | 20.16 | 42.75 | 131.96 |
| IV | 2.15 | 26.09 | 0.53 | 0.87 | 5.02 | 0.66 | 9.85 | 8.21 | 0.62 | 1.17 | 1.29 | 2.98 | 10.56 | 26.15 | 35.95 | 20.19 | 43.86 | 138.29 |
| IV | 1.93 | 26.96 | 1.00 | 0.66 | 7.33 | 0.89 | 8.02 | 7.83 | 0.56 | 0.90 | 0.99 | 2.84 | 10.12 | 27.06 | 38.25 | 18.50 | 43.25 | 154.40 |
| V | 2.17 | 27.18 | 0.66 | 0.87 | 7.05 | 0.72 | 6.66 | 7.36 | 0.80 | 1.21 | 1.34 | 3.16 | 11.20 | 25.82 | 39.04 | 16.38 | 44.58 | 136.38 |
| V | 2.33 | 24.65 | 0.57 | 1.00 | 7.11 | 0.67 | 9.13 | 8.78 | 0.82 | 1.37 | 1.53 | 2.81 | 9.93 | 25.60 | 36.82 | 20.14 | 43.04 | 133.89 |
| V | 2.35 | 26.54 | 0.47 | 0.15 | 7.73 | 0.17 | 3.78 | 8.85 | 0.68 | 1.28 | 0.96 | 3.36 | 10.67 | 29.82 | 38.46 | 13.96 | 47.59 | 148.21 |
| V | 2.04 | 25.77 | 0.56 | 0.90 | 6.50 | 0.50 | 7.78 | 7.26 | 0.75 | 1.13 | 1.21 | 3.02 | 10.56 | 28.17 | 37.02 | 17.08 | 45.89 | 144.53 |
| V | 2.13 | 26.44 | 0.69 | 0.83 | 6.71 | 0.70 | 6.97 | 7.87 | 0.92 | 1.31 | 1.45 | 3.12 | 11.05 | 26.05 | 37.83 | 17.19 | 44.98 | 125.56 |
| V | 2.33 | 25.65 | 0.56 | 0.52 | 6.97 | 0.23 | 8.49 | 7.61 | 0.97 | 1.45 | 1.31 | 3.10 | 10.93 | 26.11 | 37.20 | 17.84 | 44.96 | 140.82 |
| V | 2.21 | 25.94 | 0.39 | 0.68 | 7.10 | 0.24 | 7.32 | 7.68 | 0.62 | 1.15 | 1.11 | 3.20 | 11.08 | 27.83 | 37.52 | 16.56 | 45.91 | 140.19 |
| V | 2.49 | 21.49 | 0.55 | 0.95 | 6.88 | 0.84 | 10.24 | 8.44 | 0.73 | 1.27 | 1.42 | 2.97 | 10.49 | 27.20 | 33.74 | 21.15 | 45.11 | 136.70 |
| V | 2.02 | 26.26 | 1.14 | 0.65 | 6.86 | 0.84 | 9.48 | 7.61 | 0.47 | 0.90 | 0.99 | 3.04 | 10.82 | 25.64 | 37.37 | 19.86 | 42.77 | 139.59 |
| V | 2.03 | 25.01 | 0.56 | 0.77 | 6.68 | 0.63 | 9.26 | 8.29 | 0.74 | 1.62 | 1.76 | 2.82 | 10.21 | 26.13 | 36.14 | 19.64 | 44.22 | 137.29 |
| V | 2.03 | 26.08 | 0.67 | 0.79 | 6.63 | 0.75 | 7.94 | 8.03 | 0.67 | 0.98 | 1.06 | 2.97 | 10.45 | 27.11 | 37.45 | 18.23 | 44.32 | 152.10 |
| VI | 2.25 | 26.04 | 0.59 | 0.82 | 6.88 | 0.69 | 7.54 | 7.85 | 0.81 | 1.34 | 1.46 | 3.04 | 11.13 | 25.95 | 37.79 | 17.50 | 44.71 | 134.50 |
| VI | 2.01 | 25.87 | 0.90 | 0.78 | 6.81 | 0.74 | 7.71 | 7.74 | 0.70 | 1.69 | 1.92 | 2.84 | 10.08 | 26.79 | 37.04 | 18.03 | 44.92 | 137.96 |
| VI | 2.26 | 27.05 | 0.92 | 0.62 | 6.63 | 0.57 | 8.22 | 7.84 | 0.49 | 0.91 | 0.93 | 2.90 | 10.52 | 26.65 | 38.21 | 18.51 | 43.28 | 146.12 |
| VI | 2.13 | 26.96 | 0.88 | 0.66 | 6.25 | 0.81 | 7.78 | 7.77 | 0.66 | 1.07 | 1.19 | 3.04 | 10.78 | 26.67 | 37.60 | 18.09 | 44.31 | 135.20 |
| VI | 2.09 | 25.99 | 0.79 | 0.68 | 6.60 | 1.06 | 8.76 | 7.53 | 0.58 | 1.26 | 1.36 | 2.93 | 10.73 | 26.45 | 36.90 | 18.90 | 44.20 | 136.50 |
| VI | 2.04 | 26.48 | 0.71 | 0.72 | 7.29 | 1.01 | 7.57 | 8.31 | 0.69 | 1.04 | 1.16 | 2.87 | 10.25 | 26.62 | 38.02 | 18.51 | 43.47 | 155.84 |
| VI | 1.71 | 24.97 | 1.07 | 0.70 | 7.56 | 0.71 | 10.27 | 8.12 | 0.46 | 0.91 | 0.94 | 2.99 | 10.89 | 25.21 | 36.55 | 21.16 | 42.28 | 156.43 |
| VI | 2.00 | 26.62 | 0.94 | 0.69 | 7.03 | 0.90 | 7.76 | 7.90 | 0.61 | 1.05 | 1.15 | 2.87 | 10.15 | 26.77 | 38.00 | 18.52 | 43.48 | 137.65 |
| VI | 2.47 | 25.48 | 0.58 | 0.93 | 6.46 | 0.72 | 8.67 | 7.58 | 0.71 | 1.13 | 1.19 | 3.13 | 11.40 | 25.62 | 37.21 | 18.48 | 44.31 | 144.51 |
| VI | 2.43 | 26.36 | 0.75 | 0.72 | 5.60 | 0.78 | 9.97 | 7.28 | 0.71 | 1.43 | 1.61 | 2.79 | 9.88 | 25.77 | 36.85 | 19.93 | 43.22 | 149.73 |
| VI | 2.08 | 26.42 | 0.33 | 0.77 | 7.45 | 0.85 | 7.81 | 7.06 | 0.73 | 1.19 | 1.33 | 2.87 | 10.16 | 27.80 | 38.27 | 16.81 | 44.91 | 136.82 |
| VII | 1.75 | 23.64 | 0.84 | 0.67 | 7.14 | 0.88 | 7.71 | 8.67 | 0.62 | 1.31 | 1.43 | 3.19 | 11.26 | 26.60 | 35.14 | 19.45 | 45.41 | 128.74 |
| VII | 2.23 | 25.18 | 0.55 | 1.09 | 6.68 | 0.59 | 6.69 | 8.38 | 0.91 | 1.43 | 1.53 | 3.14 | 11.38 | 26.09 | 37.09 | 17.25 | 45.67 | 132.35 |
| VII | 1.84 | 24.91 | 0.73 | 0.88 | 5.91 | 0.56 | 5.96 | 7.51 | 0.94 | 1.32 | 1.35 | 3.19 | 10.89 | 27.43 | 36.93 | 16.14 | 46.94 | 145.79 |
| VII | 1.76 | 24.52 | 0.52 | 0.92 | 7.23 | 0.79 | 7.38 | 8.51 | 0.92 | 1.95 | 2.15 | 2.97 | 10.39 | 25.69 | 36.45 | 18.32 | 45.23 | 133.82 |
| VII | 2.16 | 21.29 | 0.58 | 0.87 | 7.53 | 0.88 | 7.02 | 8.69 | 0.94 | 1.87 | 2.11 | 3.00 | 10.57 | 28.46 | 33.74 | 18.27 | 48.00 | 124.54 |
| VII | 2.00 | 25.29 | 0.54 | 0.95 | 7.38 | 0.71 | 7.59 | 8.43 | 0.81 | 1.44 | 1.60 | 3.12 | 11.07 | 25.13 | 37.43 | 18.29 | 44.28 | 121.33 |
| VII | 2.12 | 22.07 | 0.58 | 0.87 | 7.27 | 0.95 | 8.04 | 8.15 | 0.97 | 1.26 | 1.38 | 3.07 | 10.79 | 28.61 | 34.27 | 18.62 | 47.11 | 132.37 |
| VII | 2.16 | 26.08 | 0.92 | 0.81 | 7.78 | 0.93 | 8.10 | 7.76 | 0.76 | 1.23 | 1.34 | 2.79 | 9.77 | 25.51 | 38.69 | 18.81 | 42.50 | 130.35 |
| VII | 2.61 | 20.85 | 0.92 | 0.78 | 7.14 | 0.98 | 8.04 | 8.32 | 0.78 | 1.35 | 1.50 | 3.21 | 11.35 | 28.12 | 33.29 | 19.35 | 47.36 | 129.36 |
| VII | 1.91 | 26.15 | 0.73 | 0.72 | 7.51 | 0.85 | 7.33 | 8.12 | 0.65 | 1.32 | 1.42 | 3.02 | 10.97 | 25.84 | 37.98 | 17.88 | 44.14 | 137.64 |
